# Incidence and Risk Factors for Surgical Site Infections Following Emergency Laparotomies: A Prospective Observational Study

**DOI:** 10.7759/cureus.80283

**Published:** 2025-03-09

**Authors:** Mahadeo N Garale, Ajinkya K Rewatkar, Akhilesh V Moktali, Abhay Dalvi

**Affiliations:** 1 General Surgery, Seth Gordhandas Sunderdas Medical College and King Edward Memorial Hospital, Mumbai, IND; 2 Neurosurgery, National Institute of Mental Health and Neurosciences, Bengaluru, IND; 3 General Surgery, Global Hospital, Mumbai, IND

**Keywords:** clinical outcomes, emergency exploratory laparotomy, ms general surgery, risk factors for ssi, surgical site infections (ssi)

## Abstract

Background

Surgical site infections (SSIs) are the most common healthcare-associated infections, significantly increasing postoperative morbidity, healthcare costs, and mortality. Emergency laparotomies carry a higher risk due to patient and procedural factors. This study aimed to determine the incidence, microbial patterns, and risk factors for SSIs in patients undergoing emergency laparotomies.

Methodology

A prospective, observational study was conducted at a tertiary care hospital in India from January 2018 to August 2019. Patients undergoing emergency abdominal surgeries were included. Data on patient demographics, perioperative practices, and outcomes were collected. Microbial culture and antibiotic sensitivity were analyzed for SSI cases. Statistical analysis identified significant risk factors.

Results

Of the 249 patients studied, the incidence of SSI was 17.12%, with 66% superficial, 29.5% deep, and 4.5% organ/space infections. Common pathogens included *Escherichia coli* (34%) and *Klebsiella *(15.9%). Risk factors significantly associated with SSIs included tobacco consumption (p = 0.0065), anemia (p = 0.0327), hypoproteinemia (p = 0.0016), obesity (p = 0.0030), and the use of drains (p = 0.0077).

Conclusions

SSIs remain a significant complication following emergency laparotomies. Implementing evidence-based protocols, including timely antibiotic prophylaxis and optimizing perioperative care, can reduce the burden of SSIs in resource-limited settings.

## Introduction

Surgical site infections (SSIs) are the most common hospital-acquired infections, significantly contributing to postoperative morbidity, prolonged hospitalization, and increased healthcare costs [1,2]. Despite advances in surgical techniques and infection control measures, SSIs continue to pose a major challenge in modern surgical practice. These infections occur within 30 days of surgery (or up to one year in cases involving prosthetic implants) and can affect superficial incisions, deep tissues, or organ spaces [3].

The increasing complexity of surgical procedures has contributed to a rising incidence of SSIs. Factors such as prolonged operative times, an aging population with chronic illnesses, the use of foreign implants, and immunosuppressive therapies have compounded the risks of infection [4]. Emergency laparotomies, often performed under urgent and suboptimal conditions, carry a particularly high risk due to intraoperative contamination, compromised patient physiology, and pre-existing infections [5].

SSIs are associated with significant economic and clinical burdens. Studies indicate that they can double the length of hospital stay and substantially increase healthcare costs due to additional interventions, prolonged antibiotic therapy, and intensive postoperative care [6]. Beyond financial implications, SSIs lead to increased patient discomfort, delayed recovery, and higher rates of long-term complications, including sepsis and multiorgan failure [7].

The pathogenesis of SSIs involves microbial contamination from endogenous flora or exogenous sources such as surgical instruments, healthcare personnel, or the hospital environment [8]. Common causative organisms include *Staphylococcus aureus*, *Escherichia coli*, and *Bacteroides* species, with rising concerns over multidrug-resistant pathogens [9]. Prevention strategies focus on aseptic techniques, optimal timing of prophylactic antibiotics, and careful postoperative wound management [10]. Studies suggest that administering prophylactic antibiotics within two hours before incision significantly reduces SSI risk [11].

Given the persistent burden of SSIs, this study aims to evaluate their incidence, identify the predominant causative organisms, and analyze key modifiable risk factors. The findings will contribute to evidence-based preventive strategies, ultimately improving surgical outcomes and reducing healthcare-associated infections [12].

This study was performed to prospectively determine the incidence of SSI in emergency abdominal surgeries. This study looks at emergency exploratory laparotomies performed in the emergency operating room, with the hope of paving the way for future studies in different types of operations at this and other hospitals.

This study had the following objectives: (1) to estimate the incidence of abdominal SSIs, following emergency laparotomies; (2) to correlate the microbiological organisms with postoperative SSI (superficial, deep, and organ/space); and (3) to analyze the association between various risk factors and the development of SSIs.

## Materials and methods

This study was conducted at a tertiary healthcare center in India, equipped with emergency care facilities, including dedicated setups for surgical emergencies. A prospective, observational study design was employed to evaluate SSIs in patients undergoing emergency abdominal surgeries. The study was conducted from January 2018 to August 2019.

Ethical considerations

Approval was obtained from the Institutional Ethics Committee of Seth Gordhandas Sunderdas Medical College and King Edward Memorial Hospital before initiating the study (approval number: EC/211/2018). Institutional and departmental records of patients were accessed only after obtaining the necessary permissions and authorization to ensure compliance with ethical guidelines. Written informed consent was obtained from all patients before their enrollment in the study.

Study population and criteria

The study included patients aged above 12 years who underwent emergency abdominal surgeries in the tertiary care hospital. Exclusion criteria included patients with a history of previous abdominal surgery, those with pre-existing wound infections at the surgical site, patients aged 12 years or younger, and patients who did not consent to being part of the study. Pregnant and immunocompromised patients were also excluded from the study.

Sample size calculation

The sample size was determined based on the current international SSI rate for abdominal surgeries, which is approximately 20%. The following standard formula was used for sample size estimation: N = Z^2^(1 - P) × P/ C^2^, where N is the sample size, Z is 1.96 (standard normal deviate corresponding to a 95% confidence interval), P is 20% (prevalence of abdominal wound infections), and C is 5% (absolute precision required). The sample size was calculated to be 249. However, 257 patients were recruited in this study to account for potential drop-outs. The raised sample size also increased the statistical power of this study.

Study procedure

Patients who developed SSIs and met the inclusion criteria were enrolled in the study. Two wound swabs were collected: one after paint draping and another if a patient developed an SSI postoperatively. In cases where the infection worsened, a repeat swab was taken. The collected samples were transported to the laboratory within 30 minutes for microbiological analysis.

Microbiological analysis

The collected specimens were processed using standard microbiological protocols. Bacterial cultures were performed using sheep blood agar, MacConkey’s agar, and Robertson’s cooked meat medium for anaerobes. Inoculated media were incubated under aerobic and anaerobic conditions for 24-48 hours. Additionally, a portion of the specimen was inoculated into tryptic soy broth as an enrichment medium and sub-cultured after 24 hours. Fungal cultures were performed using Sabouraud’s dextrose agar, with incubation extending up to 10 days before reporting negative growth. Species identification was conducted using standard phenotypic tests, and antibiotic sensitivity testing was performed using the Kirby-Bauer disk diffusion method. Results were interpreted following the Clinical Laboratory Standards Institute (CLSI, 2018) guidelines.

Statistical analysis

Descriptive statistical analysis was performed using measures such as mean, median, and mode. Categorical variables were analyzed using the chi-square test to determine associations between various risk factors and the occurrence of SSIs. The chi-square test was applied to assess factors such as hypertension, diabetes mellitus, anemia, body mass index (BMI), tobacco and alcohol use, and perioperative parameters such as duration of surgery and the amount of blood loss. A p-value <0.05 was considered statistically significant.

## Results

**Figure 1:**
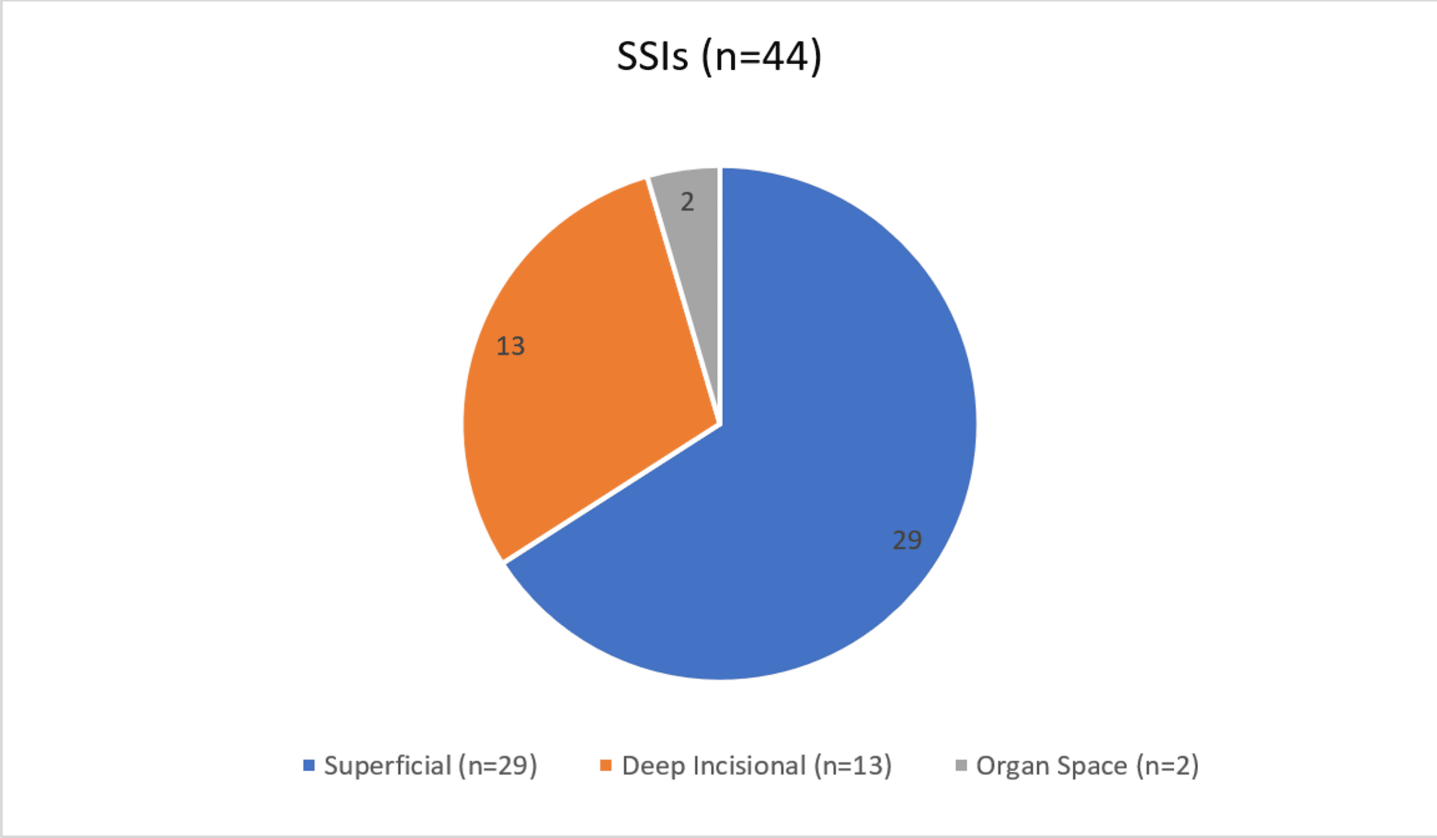
Incidence of various types of surgical site infections (SSIs).

A total of 257 patients who underwent emergency abdominal surgeries were included in this study. Overall, 44 (17.12%) patients developed SSIs and 213 (82.88%) did not. Superficial SSI (n = 29) was the most common among the three categories in our study (Figure 1).

Patient demographics

Age

**Figure 2:**
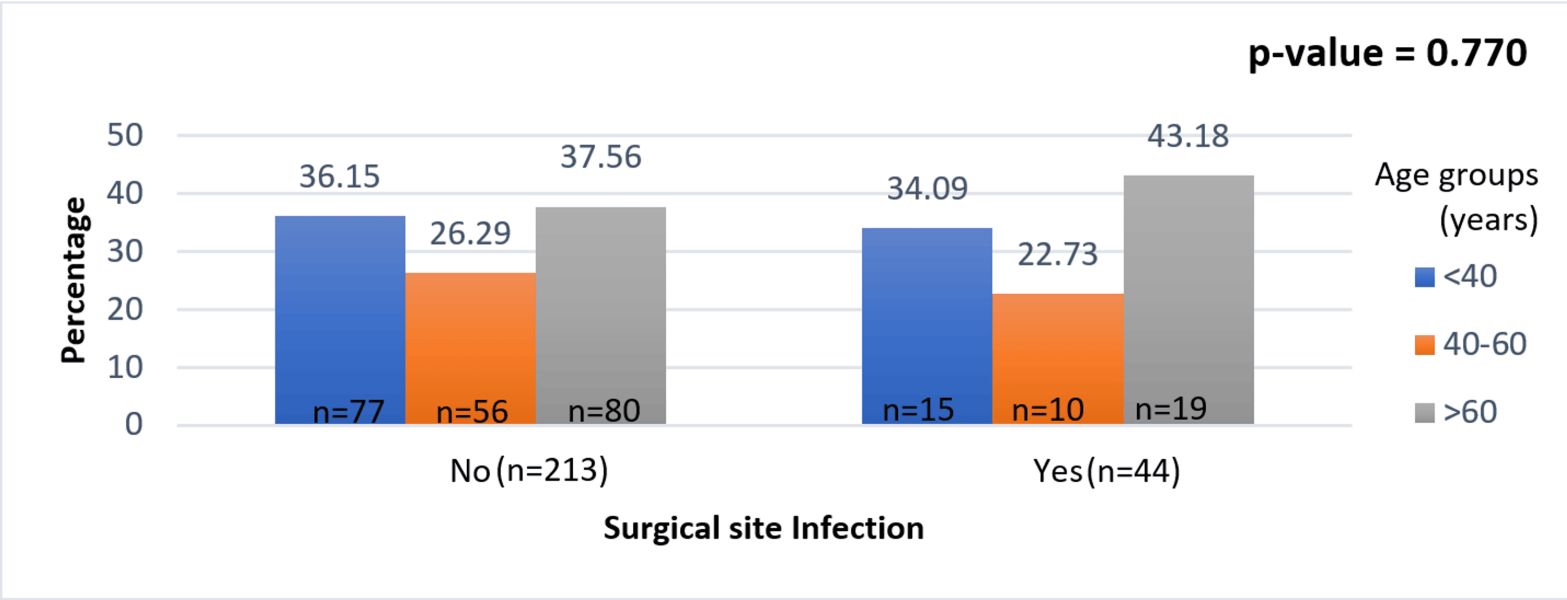
Association between age and surgical site infections (SSIs).

Among the 257 patients, the mean age was 42.6 years (±14.2). The age of the patients was grouped into three categories (<40 years, 40-60 years, and >60 years). It was found that the extremes of age were risk factors for development of SSIs (Figure 2).

Sex

**Figure 3:**
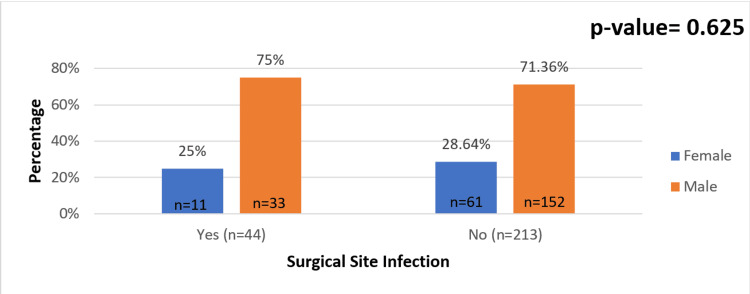
Association between sex and surgical site infections (SSIs).

Our study found no statistically significant association between the sex of patients and SSIs. However, the incidence of SSI was slightly higher in males. Overall, 71.36% of patients who did not develop SSIs were males, whereas 75% of the patients who did develop SSIs were males. The incidence of SSI in males was 17.84% (n = 33) and that of females was 15.28% (n = 11) (Figure 3).

Risk factors

Various risk factors for development of SSIs were assessed, including hypertension, diabetes mellitus, tuberculosis, tobacco consumption, alcohol consumption, use of steroid medication, anemia, obesity (BMI >25 kg/m^2^), serum albumin levels, and peptic ulceration. We found that anemia (p = 0.0327), tobacco consumption (p = 0.0065), hypoproteinemia (p = 0.0016), and being overweight (BMI >25 kg/m^2^) (p = 0.0030) yielded statistically significant associations with development of SSIs. Overall, 123 patients were anemic (hemoglobin <10 g/dL), of whom 28 patients developed SSIs. In total, 113 patients consumed tobacco in some form, of whom 28 developed SSIs. Further, 90 patients had hypoproteinemia (serum albumin <3 g/dL), of whom 25 patients developed SSIs. Finally, 106 patients were overweight (BMI >25 kg/m^2^), of whom 28 had SSIs (Table 1).

**Table 1:** Association between risk factors and surgical site infections (SSIs).

Risk factor	Surgical site infections		Chi-square value	P-value
	No (n = 213)	Yes (n = 44)		
Hypertension (27.1%)	60 (28.17%)	11 (25.00%)	0.183	0.669
Diabetes mellitus (24.12%)	52 (24.41%)	10 (22.73%)	0.057	0.812
Tuberculosis (10.89%)	23 (10.80%)	5 (11.36%)	0.012	0.913
Tobacco consumption (43.97%)	85 (39.91%)	28 (63.63%)	7.40	0.0065
Alcohol consumption (38.13%)	82 (38.50%)	16 (36.36%)	0.071	0.791
Steroid use (2.72%)	6 (2.82%)	1 (2.27%)	0.040	1.00
Anemia (hemoglobin <10 g/dL) (47.86%)	95 (44.60%)	28 (63.63%)	4.56	0.0327
Overweight (BMI >25 kg/m^2^) (41.25%)	78 (36.62%)	28 (63.63%)	11.63	0.0030
Hypoprotenemia (serum albumin <3 g/dL) (35.02%)	65 (30.52%)	25 (56.82%)	9.96	0.0016
Peptic ulceration (2.72%)	6 (2.82%)	1 (2.27%)	0.040	1.00

Operative factors

Various operative factors were assessed, including time from symptom to surgery, operative time, total blood loss, and use of drains. The use of drains was found to be associated with SSI development (p = 0.0077). Drains were placed in 215 patients (144 intra-abdominal, 71 subcutaneous), of whom 40 patients developed SSIs (34 with intra-abdominal drains, six with subcutaneous drains) (Tables 2-5). The p-values of each operative factor have been reported as a whole and not for the individual categories of the variable for ease of interpretation.

**Table 2:** Association of time to surgery with surgical site infections (SSIs). Chi-square value = 0.264; df = 2; p-value = 0.876.

Symptom to surgery	Surgical site infections		Total
	No (n = 213)	Yes (n = 44)	
<24 hours	52 (24.41)	10 (22.73)	62 (24.1)
24–48 hours	106 (49.77)	21 (47.73)	127 (49.4)
>48 hours	55 (25.82)	13 (29.55)	68 (26.5)

**Table 3:** Association of operative time with surgical site infections (SSIs). Chi-square value = 3.90; df = 2; p-value = 0.154.

Operative time	Surgical site infections		Total
	No (n = 213)	Yes (n = 44)	
<1 hour	18 (8.45)	2 (4.55)	20 (7.8)
1–2 hours	89 (41.78)	13 (29.55)	102 (39.7)
>2 hours	106 (49.77)	29 (65.91)	135 (52.5)

**Table 4:** Association of blood loss with surgical site infections (SSIs). Chi-square value = 2.62; df = 2; p-value = 0.248.

Total blood loss	Surgical site infections		Total
	No (n = 213)	Yes (n = 44)	
<150 mL	97 (45.54)	16 (36.36)	113 (44.0)
150–500 mL	83 (38.97)	17 (38.64)	100 (38.9)
>500 mL	33 (15.49)	11 (25.00)	44 (17.1)

**Table 5:** Association between drain placement and surgical site infections (SSIs). Chi-square value = 9.74; df = 2; p-value = 0.0077.

Drains placed	Surgical site infections		Total
	No (n = 213)	Yes (n = 44)	
No drain	38 (23.47)	4 (20.45)	42 (23.0)
Subcutaneous	65 (12.68)	6 (11.36)	71 (12.5)
Intra-abdominal	110 (63.85)	34 (68.18)	144 (64.6)

Association with surgeries performed

Perforation peritonitis was the most common operation performed. Overall, 18 of 115 patients operated for perforation peritonitis developed an SSI. On the other hand, gastric outlet obstruction, sigmoid volvulus, superior mesenteric vein thrombosis, and intussusception were the least common of all the diagnoses to undergo explorations during this period, each accounting for 1.6%. No surgery was significantly associated with SSIs (Table 6).

**Table 6:** Association between various surgeries and surgical site infections (SSIs). Chi-square value = 3.23; df = 8; p-value = 0.598.

Diagnosis	Surgical site infections		Total
	No (n = 213)	Yes (n = 44)	
Acute appendicitis	74 (34.74)	12 (27.27)	86 (33.5)
Acute cholecystitis	15 (7.04)	5 (11.36)	20 (7.8)
Perforation peritonitis	97 (45.54)	18 (40.91)	115 (44.8)
Gastric outlet obstruction	3 (1.41)	1 (2.27)	4 (1.6)
Splenic lacerations	3 (1.41)	1 (2.27)	4 (1.6)
Liver abscess	12 (5.63)	4 (9.09)	16 (6.2)
Sigmoid volvulus	3 (1.41)	1 (2.27)	4 (1.6)
Superior mesenteric vein thrombosis	3 (1.41)	1 (2.27)	4 (1.6)
Intussusception	3 (1.41)	1 (2.27)	4 (1.6)

Microbial patterns (preoperative)

Only four patients had a growth on preoperative swabs and all four grew *Staphylococcus* sensitive to amoxicillin, azithromycin, piperacillin-tazobactam, colistin, and linezolid. Overall, two of the four cases developed an SSI, of which one case (acute appendicitis) grew *Staphylococcus* on postoperative wound swab, and one case (acute cholecystitis) grew *Klebsiella *spp. on postoperative wound swab. The other two cases had no growth in the postoperative period and did not develop SSIs.

Microbial patterns (postoperative)

Overall, 36 (14.01%) patients reported microbial growth on postoperative swabs. Moreover, 221 (85.99%) patients demonstrated no microbial growth. All 36 patients were from the SSI group. Eight patients who developed SSIs demonstrated no microbial growth. *Escherichia coli* (34%) was the most commonly cultured organism, followed by *Klebsiella *spp. (15.9%), *Proteus *(6.8), and *Pseudomonas* (6.8%) (Table 7).

**Table 7:** Various organisms in the surgical site infection (SSI) group.

SSI present (n = 44)	Number (percentage)
Acinetobacter	2 (4.5)
Escherichia coli	15 (34.0)
Enterococcus	2 (4.5)
Klebsiella	7 (15.9)
Methicillin-resistant *Staphylococcus aureus*	1 (2.2)
Proteus	3 (6.8)
Pseudomonas	3 (6.8)
Staphylococcus	2 (4.5)
Streptococcus	1 (2.2)
No growth	8 (18.18)

The correlation between types of SSI and microbial patterns was also studied. Superficial SSI (type 1) was most commonly associated with *Escherichia coli* (20.69%) and *Klebsiella spp.* (20.69%). However, no growth was recorded in 24.14% of type 1 SSIs. Deep Incisional SSI (type 2) was most commonly associated with *Escherichia coli* (53.85%). There were only two organ space SSIs (type 3), both of which grew *Escherichia coli* (100%) (Table 8).

**Table 8:** Organisms in various types of surgical site infections (SSIs).

Organism	Superficial SSI (n = 29)	Deep incisional SSI (n = 13)	Deep organ space SSI (n = 2)
Acinetobacter	1	1	0
Escherichia coli	6	7	2
Enterococcus	1	1	0
Klebsiella	6	1	0
Methicillin-resistant *Staphylococcus aureus*	1	0	0
Proteus	2	1	0
Pseudomonas	2	1	0
Staphylococcus	2	0	0
Streptococcus	1	0	0
No growth	7	1	0

Case-wise microbiological spectrum and antibiotic sensitivity 

Acute Appendicitis

Of the 12 cases of acute appendicitis with SSIs, four yielded no growth, three were positive for *Escherichia coli*, and one each was positive for *Staphylococcus*, *Proteus*, *Klebsiella* spp., *Enterococcus*, and *Pseudomonas*. Piperacillin-tazobactam was the most sensitive of all antibiotics tested in cases of acute appendicitis with SSI.

Acute Cholecystitis

Of the five cases of acute cholecystitis with SSIs, two tested positive for *Escherichia coli*, two tested positive for *Klebsiella* spp. on final culture, and one tested positive for *Proteus*. Piperacillin-tazobactam was the most sensitive of all antibiotics tested in cases of acute cholecystitis with SSIs, and most were resistant to ceftriaxone and imipenem.

Perforation Peritonitis

Of the 18 cases of perforation peritonitis with SSIs, seven tested positive for *Escherichia coli*, two each for *Klebsiella *spp. and *Acinetobacter*, and one each for *Enterococcus*, *Pseudomonas*, methicillin-resistant *Staphylococcus aureus*, *Proteus*, and *Streptococcus*. Two cases showed no growth. Meropenem and levofloxacin were the most sensitive of all antibiotics tested in cases of perforation peritonitis with SSIs, and most organisms were resistant to imipenem.

Liver Abscess

Of the 4 cases of liver abscess with SSIs, two were positive for *Escherichia coli* and one each for *Klebsiella* spp. and *Pseudomonas*. Meropenem and piperacillin-tazobactam were the most sensitive of all antibiotics tested in cases of liver abscess with SSIs. The most resistance was seen for ampicillin, ceftazidime, and cefuroxime.

Other Surgeries with SSIs

One case of splenic laceration grew *Staphylococcus*, one case of sigmoid volvulus grew *Escherichia coli*, one case of superior mesenteric vein thrombosis grew *Klebsiella *spp. No growth was seen in either case of gastric outlet obstruction or intussusception.

## Discussion

This prospective study was conducted in a large-volume tertiary care center in a metropolitan city in India to study the incidence, rate, and risk factors associated with SSIs in our emergency department. A total of 257 patients undergoing emergency exploratory laparotomies in the years 2018-2019 were included in the study.

Classifying wounds based on the level of contamination is becoming less common in modern practice [3]. Haley’s study indicated that infection rates in clean surgical cases could range from 1% to 16% [13]. This finding led Nicolls to remark that, while not definitively proven, patient-related factors seem to play the most significant role in postoperative infections [14]. This suggests that when proper preoperative patient preparation is followed, individual patient characteristics largely determine the likelihood of developing an SSI. However, it is important to acknowledge that the extent of wound contamination still plays a role in infection risk and should not be entirely disregarded.

Incidence of abdominal SSI

The overall incidence of SSIs in abdominal surgeries observed in this study was 17.1%, which is notably higher than the 12% reported in the surgical literature [12]. However, this rate falls within the broader infection range of 2.8-17% documented in various studies. Research conducted in different regions of India has reported SSI rates ranging from 6.09% to 38.7% [15]. Comparatively, the infection rates in Indian hospitals are significantly higher than those in other countries, where SSIs occur at approximately 2.8% in the United States and between 2% and 5% in European nations [16].

Sex

The incidence of abdominal SSI was found to be 17.83% in male patients and 15.27% in female patients. However, the clinical significance of this difference remains unclear. Various studies have suggested that the following factors that may contribute to this disparity between genders: (1) Higher rates of nicotine use and alcohol consumption, which are more prevalent among men, may contribute to an increased risk of infection; (2) differences in immune response intensity between males and females could play a role; (3) gender-related variations in cytokine release have been observed, with men exhibiting higher plasma levels of pro-inflammatory mediators such as procalcitonin, interleukin-6, and tumor necrosis factor-α; (4) in contrast, women tend to have elevated levels of anti-inflammatory cytokines such as interleukin-10; (5) overall, women appear to have a stronger immunological capacity to combat septic challenges, potentially contributing to their lower SSI rates [17].

Age

The present study confirms the understanding that there is a gradual rise in the incidence of wound infections as age advances. The incidence showed a gradual rise from 16.3% in the <40-year age groups to 19.2% in patients aged more than 60 years. Likewise, Cruse and Foord observed in their study that older patients are more likely to develop infections in clean wounds than younger patients [18]. Similar findings were demonstrated by Mead et al., who observed an increased wound infection in patients less than one year old (2.7%) or greater than 50 years old (2.8%) versus those 1 to 50 years old (0.7%).

The high SSI incidence of 19.2% in patients above 60 years in our study is perhaps due to decreased immunocompetence and increased chances of co-morbid factors, such as diabetes mellitus, hypertension, and chronic ailments, such as asthma and arthritis, conditions requiring steroid therapy, along with personal habits such as smoking and alcoholism. Although age is an immutable factor, its role as a risk factor for wound infection appears to be modest at best.

Anemia, hypoproteinemia, diabetes mellitus, obesity

Incidence of SSIs among various risk factors was anemia at 22.76%, hypoproteinemia at 27.78%, diabetes mellitus at 16.13%, and BMI >25 kg/m^2^ at 26.41%. Similar trends have been reported in other studies [15]. These elevated infection rates may be attributed to factors such as impaired immune function, delayed wound healing, hyperglycemia, and the presence of pre-existing infections.

Drain use

The use of drains had an infection rate of 17.67% in our study. Kamat et al in 2007 reported that patients with postoperative drains were 5.8 (2.33-14.66) times more likely to develop an SSI compared to those without a drain [19]. While the proportion of those with a postoperative drain acquiring SSIs was 62.5% (15/24), it was 22.2% (20/90) among those without the drain (chi-square = 14.448, p = 0.000). Further, the infection rate increased with the increasing duration of the drain. This may be due to the nature of the surgery requiring drainage, the drain serving as a potential entry point for pathogens, or the direct impact of the drain itself on the surgical site. The nature of the surgery, the pathology itself, and the patient’s preoperative status are potential confounders in the analysis of drain usage. A multivariate regression analysis would be better suited to analyze the cause-effect relationship in future studies.

Antibiotic prophylaxis

In this study, all patients received prophylactic antibiotics, with ceftriaxone being the most common antibiotic used. On average, antibiotics were administered one to two hours before surgery. Razavi et al. in 2005 suggested that administering prophylactic antibiotics approximately 30 minutes before surgery yields the best outcomes and the lowest incidence of SSIs [20]. However, there remains an ongoing debate regarding the optimal duration and choice of antibiotics. Overall, most studies support the use of one to three intravenous doses of a second-generation cephalosporin, with or without metronidazole, ensuring that the first dose is given before the surgical incision.

Organisms isolated

In this study, *Escherichia coli *was the most frequently isolated organism, accounting for 34% of cases, followed by *Klebsiella* spp. (15.9%), *Proteus* (6.8%), and *Pseudomonas* (6.8%). Similar trends have been reported in previous research, such as the study by Kamat et al. (2008), which found that 79.33% of isolates were gram-negative bacteria, with *Pseudomonas* being the most common, followed by *Staphylococcus pyogenes *[19]. Other studies, such as that by Bo et al. (2009) in Lagos, Nigeria, also identified *Pseudomonas* as the predominant isolate [21].

The predominance of gram-negative bacilli in this study aligns with findings from other researchers. In most cases of SSIs, the causative organism originates from the patient’s endogenous flora. During abdominal surgeries, exposure of the gastrointestinal tract increases the likelihood of contamination with coliforms and other gram-negative bacilli, which was consistent with our observations. These organisms are often endemic in hospital environments, spreading easily through contaminated surfaces and medical equipment. Additionally, they exhibit high resistance to common antiseptics, making them difficult to eliminate and contributing significantly to hospital-acquired infections.

Antibiotic sensitivity and resistance

The pattern of various organisms in some of the commonly performed surgeries in our setup and their sensitivity to antibiotics was studied. Laparotomy for perforation peritonitis was the most common operation performed at 44.8%. Overall, 18 of 115 operated for perforation peritonitis had SSIs. On the other hand, gastric outlet obstruction, sigmoid volvulus, superior mesenteric vein thrombosis, and intussusception undergoing explorations developed the least SSIs during this period, each accounting for 1.6%.

Most cases of acute appendicitis were found to have SSI with *Escherichia coli*, most being sensitive to piperacillin-tazobactam. SSIs in operated cases of acute cholecystitis grew *Escherichia coli* and *Klebsiella *spp. in similar numbers and their sensitivity pattern was in favor of piperacillin-tazobactam. Imipenem was found to have the most resistance of all the antibiotics tested.

Perforation peritonitis rightly corresponded to *Escherichia coli *being the most common contaminant, as it is the most common in the gut flora. Meropenem and levofloxacin were the most sensitive of all antibiotics tested in cases of perforation peritonitis with SSIs, and most were resistant to imipenem.

Most cases of liver abscess were positive for *Escherichia coli*. Meropenem and piperacillin-tazobactam were the most sensitive of all antibiotics tested in cases of liver abscess with SSIs, and most were resistant to ampicillin, ceftazidime, and cefuroxime.

A study conducted by Akinkunmi et al. on the microbial patterns in surgical wound infections at a Nigerian hospital found that bacterial pathogens were present in all specimens. *Staphylococcus aureus* was the most frequently isolated organism, accounting for 18.3% (23 out of 126 isolates). Other notable isolates included *Pseudomonas aeruginosa* (11.1%), *Bacillus* spp. (11.1%), *Escherichia coli* (10.3%), *Candida* spp. (8.7%), *coagulase-negative Staphylococci* (8.7%), *Pseudomonas* spp. (6.3%), *Serratia odorifera *(4.7%), *Bacteroides* (4.0%), and *Enterococcus* spp. (3.2%), with the remaining isolates belonging to various *Enterobacteriaceae* species [22]. Antibiotic susceptibility testing revealed high resistance levels, with over 98% of bacterial isolates resistant to β-lactam antibiotics. Additionally, more than 70% of isolates showed resistance to erythromycin, fusidic acid, and tobramycin. In contrast, Kamat et al. (2008) found that 21.4% of *Pseudomonas* spp. isolates were sensitive to the cefoperazone-sulbactam combination, highlighting variations in bacterial resistance patterns across different studies and healthcare settings.

Study limitations

A single-center study makes it difficult to generalize our results. The use of chi-square tests without multivariate analysis does not fully adjust for confounding factors, making it difficult to assess the independent effect of risk factors such as drain use on SSIs. Another limitation is that microbial patterns may differ across regions. A limited sample size may fail to reproduce significant results for some risk factors, as demonstrated in other studies.

## Conclusions

The incidence of SSIs in this study was 17.1%. The majority of patients affected were above 60 years of age, followed by those under 40 years, indicating that extremes of age were more commonly involved in emergency surgeries at this center. While the infection rate was slightly higher in males, the difference was not statistically significant. Among the different types of SSIs, superficial infections were the most frequently observed. Key risk factors identified in this study included BMI >25 kg/m^2^ (overweight), anemia, tobacco use, and hypoproteinemia, all of which contributed to an increased susceptibility to infection. Additionally, the use of surgical drains was linked to a higher incidence of SSIs, emphasizing the need for strict aseptic precautions, particularly when using prosthetic materials and drains. *Escherichia coli* was the most commonly isolated pathogen from infected surgical wounds. Among patients undergoing emergency exploratory laparotomies, perforation peritonitis was the leading cause of SSI. These findings highlight the importance of meticulous surgical technique, infection control measures, and targeted prophylactic strategies to reduce the burden of SSIs in emergency surgical settings. Piperacillin-tazobactam and meropenem were the most sensitive drugs of all drugs tested across all cases and cultured organisms. Hence, early use of piperacillin-tazobactam or meropenem is advocated in emergency laparotomies, followed by a culture-based approach. Imipenem demonstrated the highest resistance, particularly in infections such as perforation peritonitis and acute cholecystitis, indicating its limited effectiveness against the predominant pathogens in these cases. SSIs are a big burden for patients as well as the institution in terms of morbidity, hospital stay and costs incurred. Therefore, as it always has been, “prevention is better than cure.”

## Figures and Tables

**Figure 4:**
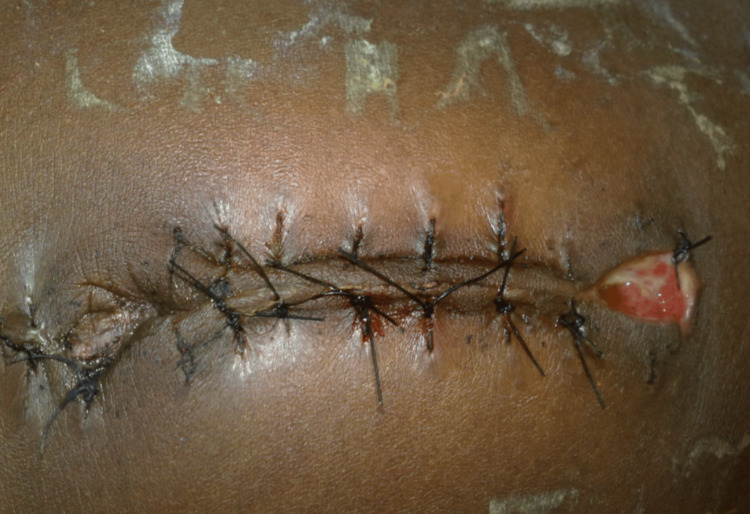
Superficial surgical site infection.

**Figure 5:**
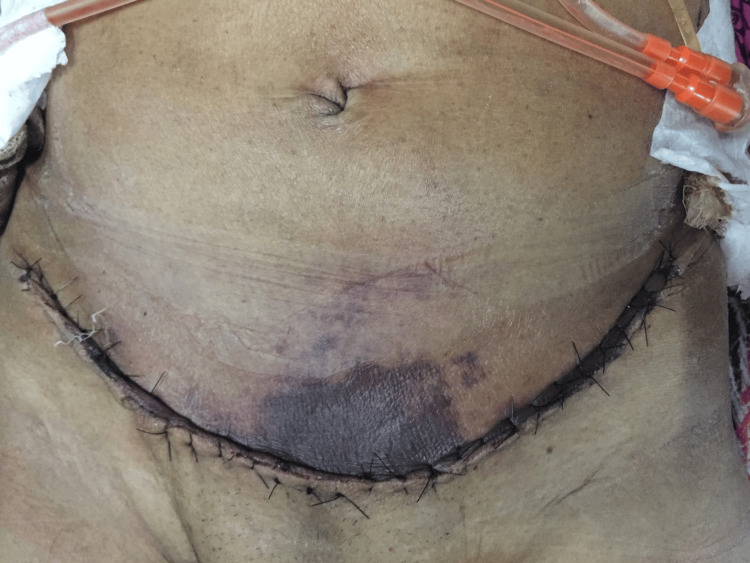
Superficial surgical site infection.

**Figure 6:**
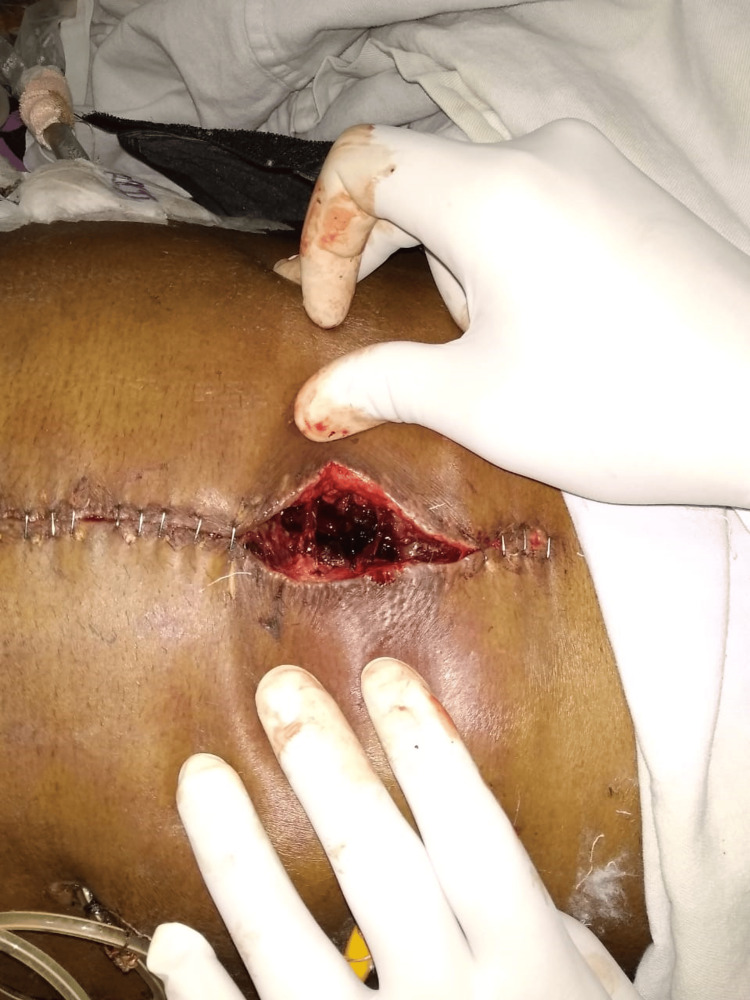
Deep incisional surgical site infection.

**Figure 7:**
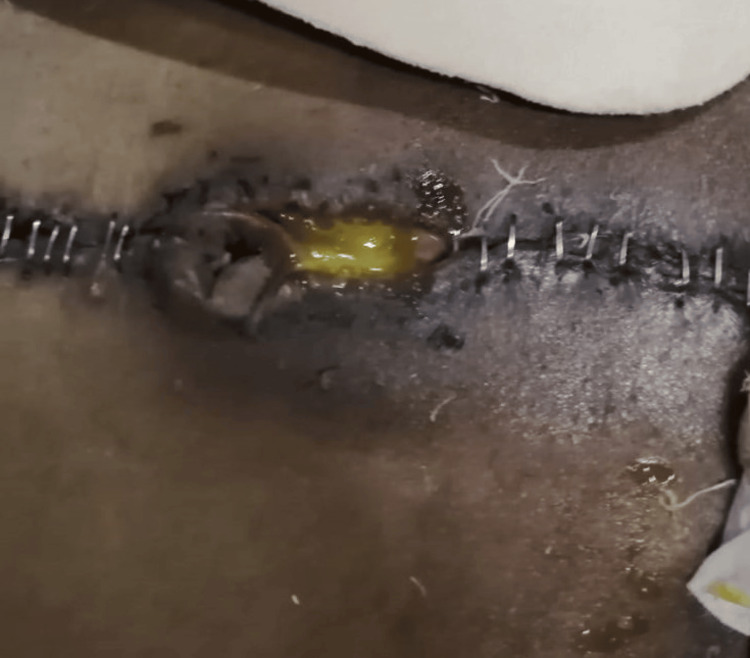
Organ space surgical site infection.

## Appendices

Appendix 1: Drug sensitivities in various surgeries from the SSI group

**Table 9:** Drug susceptibilities in operated cases of acute appendicitis. Acute appendicitis (12): no growth (4), *Escherichia coli* (3), *Staphylococcus* (1), *Proteus* (1), *Klebsiella* (1), *Enterococcus* (1), *Pseudomonas* (1).

	Sensitive, n	Resistant, n	Not tested, n
Penicillin		1	11
Amoxiclav			12
Azithromycin			12
Erythromycin		1	11
Co-trimoxazole	2		10
Methicillin	1		11
Vancomycin	1		11
Teicoplanin	1		11
Chloramphenicol		1	11
Tetracycline	1	1	10
Meropenem	2	2	8
Imipenem	3	3	6
Aztreonam	2		10
Ampicillin	1	4	7
Netilmicin	3	1	8
Colistin	4	1	7
Ceftazidime		3	9
Ceftriaxone		3	9
Ticarcillin + clavulanic acid			12
Linezolid	1		11
Clindamycin			12
Cefuroxime	1	2	9
Cefotaxime			12
Cefepime	1	3	8
Gentamycin	4		8
Amikacin	3	1	8
Ciprofloxacin		4	8
Ofloxacin			12
Levofloxacin	1	4	7
Doxycycline			12
Piperacillin + tazobactam	7		5
Polymyxin B		1	11

**Table 10:** Drug susceptibilities in operated cases of acute cholecystitis. Acute cholecystitis (5): *Escherichia coli* (2), *Klebsiella* (2), *Proteus* (1).

	Sensitive, n	Resistant, n	Not tested, n
Penicillin			5
Amoxiclav			5
Azithromycin			5
Erythromycin			5
Co-trimoxazole			5
Methicillin			5
Vancomycin		1	4
Teicoplanin			5
Chloramphenicol		1	4
Tetracycline		1	4
Meropenem	2	2	1
Imipenem		5	
Aztreonam			5
Ampicillin		2	3
Netilmicin	2	1	2
Colistin	2	1	2
Ceftazidime		3	2
Ceftriaxone		5	
Ticarcillin + clavulanic acid			5
Linezolid			5
Clindamycin		1	4
Cefuroxime		2	3
Cefotaxime		2	3
Cefepime		2	3
Gentamycin	2		3
Amikacin	2	2	1
Ciprofloxacin		2	3
Ofloxacin		1	4
Levofloxacin		3	2
Doxycycline			5
Piperacillin + tazobactam	4	1	
Polymyxin B			5

**Table 11:** Drug susceptibilities in operated cases of perforation peritonitis. Perforation peritonitis (18): *Escherichia coli* (7), *Klebsiella* (2), *Acinetobacter* (2), *Enterococcus* (1), *Pseudomonas* (1), methicillin-resistant *Staphylococcus aureus* (1), *Proteus* (1), *Streptococcus* (1), no growth (2).

	Sensitive, n	Resistant, n	Not tested, n
Penicillin		3	13
Amoxiclav	1	1	14
Azithromycin			16
Erythromycin	2	1	13
Co-trimoxazole	4		12
Methicillin	1	1	14
Vancomycin	1		15
Teicoplanin			16
Chloramphenicol			16
Tetracycline	1		15
Meropenem	10	2	4
Imipenem	1	10	5
Aztreonam	1	2	13
Ampicillin		4	12
Netilmicin	5	1	10
Colistin	7		9
Ceftazidime		4	12
Ceftriaxone		3	13
Ticarcillin + clavulanic acid			16
Linezolid	3		13
Clindamycin	1	2	13
Cefuroxime		7	9
Cefotaxime		7	9
Cefepime		6	10
Gentamycin	3	3	10
Amikacin	7	2	7
Ciprofloxacin	1	5	10
Ofloxacin		1	15
Levofloxacin	10		6
Doxycycline			16
Piperacillin + tazobactam	9	3	4
Polymyxin B	2		14

**Table 12:** Drug susceptibilities in operated cases of liver abscess. Liver abscess (4): *Escherichia coli* (2), *Klebsiella* (1), *Pseudomonas* (1).

	Sensitive, n	Resistant, n	Not tested, n
Penicillin			4
Amoxiclav			4
Azithromycin			4
Erythromycin			4
Co-trimoxazole			4
Methicillin			4
Vancomycin			4
Teicoplanin			4
Chloramphenicol			4
Tetracycline			4
Meropenem	4		
Imipenem	3		1
Aztreonam			4
Ampicillin	1	2	1
Netilmicin	3		1
Colistin		1	3
Ceftazidime	1	2	1
Ceftriaxone	2	1	1
Ticarcillin+ clavulanic acid			4
Linezolid			4
Clindamycin			4
Cefuroxime		2	2
Cefotaxime		2	2
Cefepime	2		2
Gentamycin	1	1	2
Amikacin	2		2
Ciprofloxacin	2		2
Ofloxacin			4
Levofloxacin	3	1	
Doxycycline			4
Piperacillin + tazobactam	4		
Polymyxin B			4

**Table 13:** Drug susceptibilities in operated cases of splenic laceration. Splenic laceration (1): *Staphylococcus* (1).

	Sensitive, n	Resistant, n	Not tested, n
Penicillin		1	
Amoxiclav	1		
Azithromycin			1
Erythromycin	1		
Co-trimoxazole		1	
Methicillin	1		
Vancomycin	1		
Teicoplanin			1
Chloramphenicol			1
Tetracycline	1		
Meropenem			1
Imipenem			1
Aztreonam			1
Ampicillin			1
Netilmicin	1		
Colistin			1
Ceftazidime			1
Ceftriaxone			1
Ticarcillin + clavulanic acid			1
Linezolid			1
Clindamycin	1		
Cefuroxime			1
Cefotaxime			1
Cefepime			1
Gentamycin	1		
Amikacin			1
Ciprofloxacin			1
Ofloxacin			1
Levofloxacin			1
Doxycycline			1
Piperacillin + tazobactam			1
Polymyxin B			1

**Table 14:** Drug sensitivities in the operated case of sigmoid volvulus. Sigmoid volvulus (1): *Escherichia coli* (1).

	Sensitive, n	Resistant, n	Not tested, n
Penicillin			1
Amoxiclav			1
Azithromycin			1
Erythromycin			1
Co-trimoxazole			1
Methicillin			1
Vancomycin			1
Teicoplanin			1
Chloramphenicol		1	
Tetracycline			1
Meropenem	1		
Imipenem	1		
Aztreonam			1
Ampicillin			1
Netilmicin			1
Colistin	1		
Ceftazidime	1		
Ceftriaxone	1		
Ticarcillin + clavulanic acid			1
Linezolid		1	
Clindamycin			1
Cefuroxime		1	
Cefotaxime		1	
Cefepime		1	
Gentamycin			1
Amikacin	1		
Ciprofloxacin			1
Ofloxacin			1
Levofloxacin			1
Doxycycline			1
Piperacillin + tazobactam	1		
Polymyxin B			1

**Table 15:** Drug susceptibilities in the operated case of superior mesenteric vein thrombosis. Superior mesenteric vein thrombosis (1): *Klebsiella* (1).

	Sensitive, n	Resistant, n	Not tested, n
Penicillin			1
Amoxiclav			1
Azithromycin			1
Erythromycin			1
Co-trimoxazole			1
Methicillin			1
Vancomycin			1
Teicoplanin			1
Chloramphenicol			1
Tetracycline			1
Meropenem		1	
Imipenem		1	
Aztreonam			1
Ampicillin		1	
Netilmicin			1
Colistin	1		
Ceftazidime			1
Ceftriaxone			1
Ticarcillin + clavulanic acid			1
Linezolid			1
Clindamycin			1
Cefuroxime			1
Cefotaxime		1	
Cefepime		1	
Gentamycin		1	
Amikacin		1	
Ciprofloxacin			1
Ofloxacin			1
Levofloxacin		1	
Doxycycline			1
Piperacillin + tazobactam		1	
Polymyxin B			1

Appendix 2: Types of SSI
